# Ultra-High-Resolution Photon-Counting Detector CT Arthrography of the Ankle: A Feasibility Study

**DOI:** 10.3390/diagnostics13132201

**Published:** 2023-06-28

**Authors:** Karsten Sebastian Luetkens, Jan-Peter Grunz, Andreas Steven Kunz, Henner Huflage, Manuel Weißenberger, Viktor Hartung, Theresa Sophie Patzer, Philipp Gruschwitz, Süleyman Ergün, Thorsten Alexander Bley, Philipp Feldle

**Affiliations:** 1 Department of Diagnostic and Interventional Radiology, University Hospital Würzburg, Oberdürrbacher Straße 6, 97080 Würzburg, Germany; 2 Department of Orthopaedic Surgery, University of Würzburg, König-Ludwig-Haus, Brettreichstr. 11, 97074 Würzburg, Germany; 3 Institute of Anatomy and Cell Biology, University of Würzburg, Koellikerstraße 6, 97070 Würzburg, Germany

**Keywords:** photon-counting CT, arthrography, ankle, cartilage, radiation dosage

## Abstract

This study was designed to investigate the image quality of ultra-high-resolution ankle arthrography employing a photon-counting detector CT. Bilateral arthrograms were acquired in four cadaveric specimens with full-dose (10 mGy) and low-dose (3 mGy) scan protocols. Three convolution kernels with different spatial frequencies were utilized for image reconstruction (ρ_50_; Br98: 39.0, Br84: 22.6, Br76: 16.5 lp/cm). Seven radiologists subjectively assessed the image quality regarding the depiction of bone, hyaline cartilage, and ligaments. An additional quantitative assessment comprised the measurement of noise and the computation of contrast-to-noise ratios (CNR). While an optimal depiction of bone tissue was achieved with the ultra-sharp Br98 kernel (*S* ≤ 0.043), the visualization of cartilage improved with lower modulation transfer functions at each dose level (*p* ≤ 0.014). The interrater reliability ranged from good to excellent for all assessed tissues (intraclass correlation coefficient ≥ 0.805). The noise levels in subcutaneous fat decreased with reduced spatial frequency (*p* < 0.001). Notably, the low-dose Br76 matched the CNR of the full-dose Br84 (*p* > 0.999) and superseded Br98 (*p* < 0.001) in all tissues. Based on the reported results, a photon-counting detector CT arthrography of the ankle with an ultra-high-resolution collimation offers stellar image quality and tissue assessability, improving the evaluation of miniscule anatomical structures. While bone depiction was superior in combination with an ultra-sharp convolution kernel, soft tissue evaluation benefited from employing a lower spatial frequency.

## 1. Introduction

The tibiotalar cartilage bears up to five times the body’s weight [1], posing a risk factor for osteoarthritis, especially if these forces increase [2]. At the same time, traumatic osteochondral lesions and ligament injuries of the ankle, as well as resulting unphysiological load distributions, are frequent [3,4,5]; thus, assessing the stability of chondral lesions represents a crucial diagnostic imaging task, as chondral delamination and subchondral pathologies may not be visible in direct arthroscopy but can impact therapeutic concepts [6,7].

Magnetic resonance imaging (MRI) is widely recognized as the reference standard for cross-sectional imaging of soft tissue pathologies [8,9]; however, as the ankle’s cartilage consists of only a thin hyaline chondral layer, averaging 1.1 mm (range 0.4–2.1 mm) [10,11,12,13], the depiction of discreet injuries continues to pose a challenge in MRI with reported sensitivities as low as 50% at 1.5 T and 75% at 3.0 T for osteochondral ankle injuries [12,14]. On the other hand, computed tomography (CT) arthrography represents a powerful, well-established, and preferable alternative for discerning osteochondral lesions, e.g., for the elbow [15]. Comparing CT to MR arthrography of the ankle joint at 1.0 and 1.5 T with regards to cartilage lesions, Schmid et al. reported a superior level of observer agreement and reliability favoring the former [10]. Similarly, Pöhler et al. demonstrated an advantage for CT arthrography versus 3.0 T MR arthrography regarding the assessment of lesion depth in artificially induced osteochondral lesions of the talar dome while maintaining a comparable accuracy [16].

Recently, the emergence of photon-counting detector CT (PCD-CT) catalyzed further advances in depicting minute structures with unsurpassed radiation dose efficiency for CT thus far [17,18]. PCD architecture eliminates the need for a two-step conversion process of incoming X-ray photons as opposed to the current energy-integrating detector systems [19,20]. As the generated electric impulses are proportional to every photon’s particular energy above a certain threshold, low-energy photons are no longer down-weighted, and contrast-to-noise ratios are significantly improved [21,22]. The current and first PCD-CT generation allows for an in-plane resolution of as little as 0.11 mm in ultra-high-resolution (UHR) mode without dose penalty, facilitating the visualization of microstructures in particular [23]. 

While numerous studies have analyzed the impact of PCD technology on bone imaging [24,25], to the authors’ best knowledge, no thorough investigation was conducted regarding PCD-CT arthrography thus far. Aiming to address the current research gap, this study evaluates the feasibility of ankle arthrograms with the novel detector technology, establishing a clinically reproducible scan protocol in the process. We hypothesized that the PCD-CT arthrography with UHR collimation would aid the assessment of minuscule anatomical structures, such as thin cartilage layers and ligamentous stabilizers.

## 2. Materials and Methods

### 2.1. Cadaveric Specimens

The anatomical institute of the local university allocated four fresh-frozen, non-formalin-fixated cadaveric specimens to the radiology department for this investigation. During their respective lifetimes, the body donors had consented to posthumous use of their remains for study and research purposes. No additional selection criteria were imposed. The institutional review board of our university waived the need for further written informed consent and granted permission for this study.

### 2.2. Arthrography Procedure

A board-certified radiologist with nine years of experience in musculoskeletal imaging performed bilateral ankle arthrographies in all four cadaveric specimens. Using an ultrasound for guidance (Acuson Sequoia, Siemens Healthineers, Erlangen, Germany), each tibiotalar joint was infiltrated with a 20-gauge needle (Sterican^®^, Braun SE, Melsungen, Germany) on the medial side of the anterior tibial tendon in analogy to a clinical procedure. A combination of 50% iodinated contrast agent (Imeron 300^®^, Bracco S.p.A., Milan, Italy) and 10 mg per ml of a local anesthetic (Mecain^®^, Puren Pharma GmbH & Co. KG, Munich, Germany) was injected via the articular access. The injection volume was selected as high as feasible, ranging between 7 and 9 mL. Using fresh-frozen specimens instead of formalin-fixated cadavers allowed for realistic tissue properties and, subsequently, an overall procedure representative of ankle arthrograms in vivo. No particular challenges or limitations were encountered during the arthrography procedures.

### 2.3. Image Acquisition and Reconstruction Parameters

Directly following injection, the ankle joints were scanned using a first-generation cadmium-telluride-based PCD-CT system (Naeotom Alpha, Siemens Healthineers). All examinations were performed in UHR scan mode using a collimation of 120 × 0.2 mm. With tube potential set to 120 kVp, a full-dose and low-dose scan were acquired in each specimen with effective tube currents of 125 and 38 mAs. Resulting volume CT dose indices (CTDI_vol_) amounted to 10.0 and 3.0 mGy. Detailed scan parameters are provided in Table 1. Ankles were individually scanned and reconstructed in three standardized orientations (axial, coronal, and sagittal) with an increment of 0.6 mm and a field of view of 100 mm. Matrix parameters were selected automatically to obtain optimal settings; thereby, the sharpest available non-UHR kernel (Br76), a medium-sharp UHR kernel (Br84), as well as the sharpest UHR kernel (Br98) were employed (Table 2). Preset window settings were 1400 and 300 HU (window width and center); however, observers were permitted to modify these according to personal preferences.

### 2.4. Subjective Image Evaluation

Seven radiologists with four to nine years of experience in musculoskeletal imaging evaluated all datasets independently using certified diagnostic monitors (RadiForce RX660, EIZO, Hakusan, Japan) in combination with standard clinical PACS software (Merlin 7.0.226222, Phönix-PACS, Freiburg, Germany). Readers were blinded to all protocol-related information. After determining whether images were suitable for diagnostic use in dichotomous fashion, observers were separately tasked with grading the image quality for bone, cartilage, and ligaments, employing a seven-point rating scale (1 = very poor; 2 = poor; 3 = fair; 4 = satisfactory; 5 = good; 6 = very good; 7 = excellent).

### 2.5. Objective Image Evaluation

Normed regions of interest were placed in the talus, talar cartilage, posterior tibiotalar ligament, and subcutaneous fat, noting mean density and standard deviation thereof. Due to its homogeneous texture, the standard deviation within subcutaneous fat was defined as image noise. Individual contrast-to-noise ratios were calculated for osseous tissue (CNR_Bone_), cartilage (CNR_Cartilage_), and ligaments (CNR_Ligament_) with the following formula:

$$CNR=\frac{mean\;attenuation\;\left(bone/cartilage/ligament\right)-mean\;attenuation\;\left(fat\right)\;}{standard\;deviation\;\left(fat\right)}\;$$

### 2.6. Statistical Analysis

All statistical analyses were performed with dedicated software (SPSS Statistics Version 28, IBM, Armonk, NY, USA). For evaluating normal distribution in continuous variables, Kolmogorov–Smirnov tests were conducted. Categorical variables were reported as absolute and relative frequencies, with median values and 10–90 percentile ranges, while normally distributed metric data were presented as means ± standard deviations. Mean rank distribution in paired non-parametric variables was assessed comparatively using Friedman tests and Bonferroni-corrected pairwise post-hoc analyses. Null hypotheses were rejected, and statistical significance was assumed if computed *p*-values were not greater than 0.05. Interrater agreement was tested using the intraclass correlation coefficient (ICC) for absolute agreement in a two-way random effects model. Following Koo and Li [26], ICC scores were interpreted as being associated with poor (<0.50), moderate (0.50–0.75), good (0.75–0.90), or excellent (>0.90) reliability.

## 3. Results

### 3.1. Subjective Image Quality Assessment

**Figure 1:**
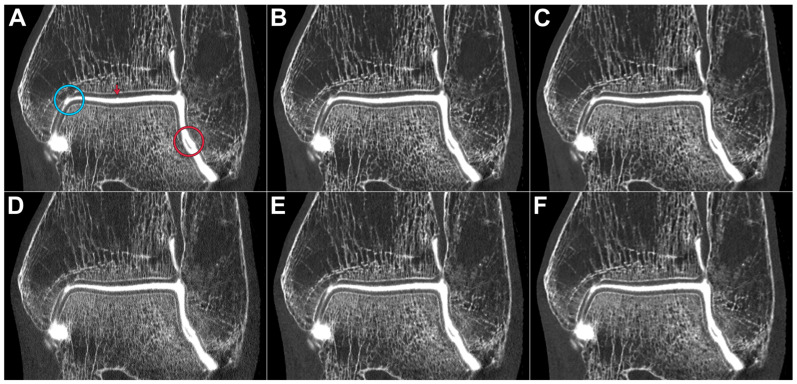
Photon-counting arthrographies in a cadaveric specimen performed with the full-dose (10 mGy; **A**–**C**) and low-dose scan protocol (3 mGy; **D**–**F**). Reconstructions were performed with the ultra-sharp kernel Br98 (**A/D**), medium UHR kernel Br84 (**B/E**), and non-UHR kernel Br76 (**C/F**). Please note the full thickness cartilage defect at the lateral aspect of the talus (**red circle**) versus the pseudodefect of the tibial plafond known as the “Notch of Harty” (**blue circle**). A superficial cartilage lesion of the central tibia is better visualized using means of full-dose arthrography (**red arrow**).

**Figure 2:**
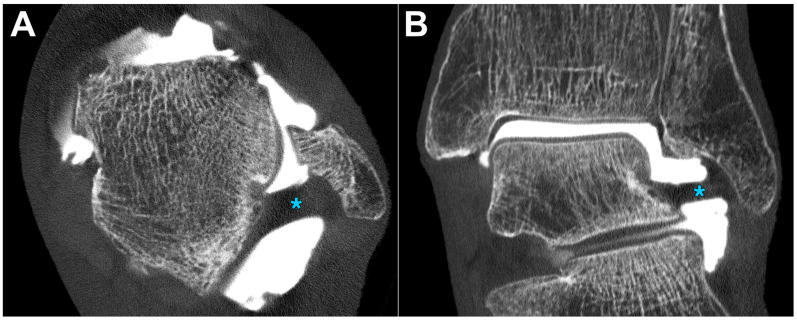
Depiction of an intact posterior tibiofibular ligament (**blue asterisk**) in axial (**A**) and coronal orientation (**B**). Photon-counting CT arthrography was performed with a CTDI_vol_ of 10 mGy. The acquired dataset was reconstructed with the ultra-sharp Br98 kernel, which possesses the highest spatial frequency of all convolution kernels available for ultra-high-resolution imaging on a photon-counting detector.

**Figure 3:**
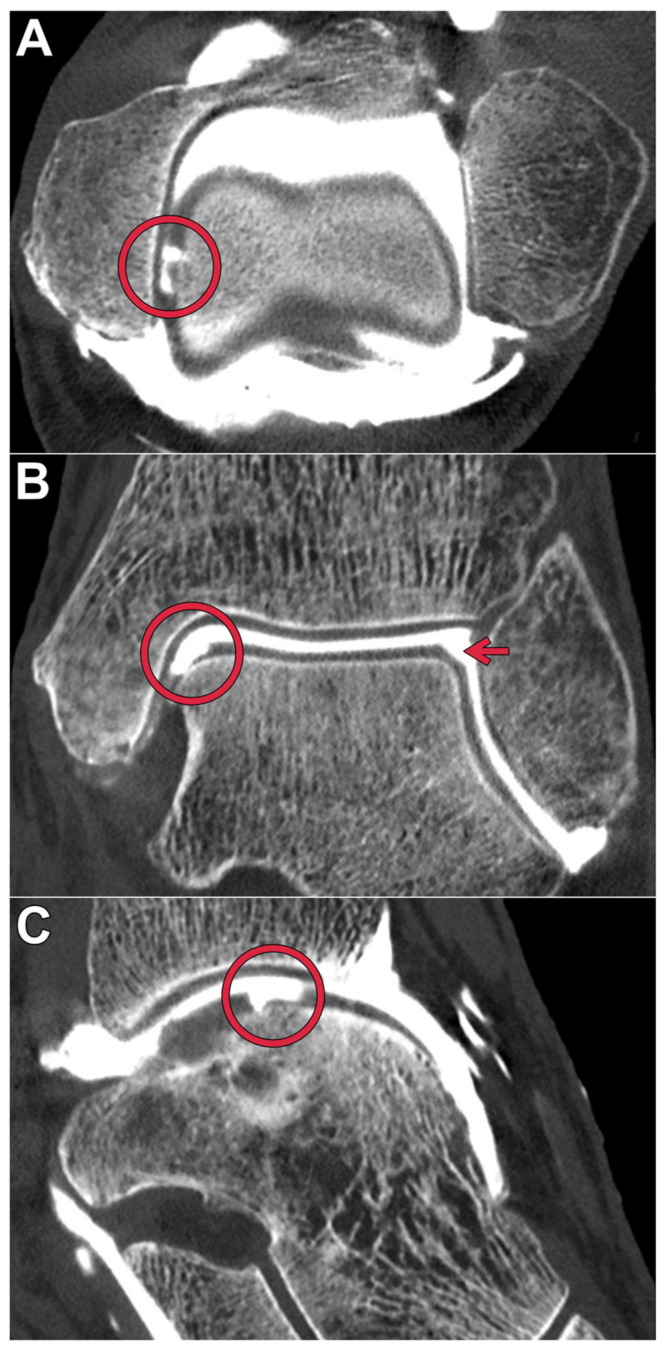
Tri-planar reformatting of a full-dose photon-counting CT arthrogram in axial (**A**), coronal (**B**), and sagittal orientation (**C**) using the medium BR84 kernel displays an osteochondral lesion of the medial talus shoulder (**red circle**). Additionally, a partial thickness cartilage injury of the lateral talus shoulder can be diagnosed (**red arrow**).

All datasets were deemed suitable for diagnostic assessment in a clinical routine by each of the observers. Table 3 summarizes the pooled image quality scores assigned for bone, cartilage, and ligaments. The optimal depiction of bone tissue was achieved in full-dose scans with the ultra-sharp Br98 reconstruction kernel (median value 7, range 6–7). All full-dose datasets were rated superior to the respective low-dose scans (*p* < 0.001). Figure 1 includes a side-by-side comparison of the six employed acquisition–reconstruction combinations, while highlighting hyaline cartilage lesions of various degrees. In contrast to osseous tissue, the assessment of cartilage benefited from applying reconstruction kernels with a lower spatial frequency within each dose level (*p* ≤ 0.014). No significant difference was ascertained between full-dose Br98 versus low-dose Br84 and Br76 reconstructions for hyaline cartilage (*p* ≥ 0.186) and ligaments *(p* ≥ 0.283). Figure 2 illustrates the depiction of an intact posterior tibiofibular ligament. A pairwise comparison matrix comprising all assessed combinations of scan protocol and reconstruction settings is displayed in Table 4. Interrater reliability for bone microarchitecture visualization was excellent, indicated with an ICC of 0.938 (95% confidence interval 0.902–0.962; *p* < 0.001), while observer agreement was good for judging cartilage (0.887; 0.779–0.940; *p* < 0.001) and ligaments (0.805; 0.661–0.889; *p* < 0.001). Figure 3 exemplifies a full thickness defect located at the medial talar shoulder and a partial thickness lesion on the lateral side.

### 3.2. Objective Image Quality Assessment

Noise levels in subcutaneous fat decreased with reduced modulation transfer function (*p* < 0.001). With regards to CNR, the non-UHR Br76 kernel superseded both assessed UHR kernels based on measurements in bone, cartilage, and ligaments (*p* < 0.007). No dose-dependent difference was ascertained for any of the tissues with Br98 (*p* > 0.999); moreover, low-dose Br76 matched the quantitative metrics of full-dose Br84 (*p* > 0.999) and even superseded Br98 (*p* < 0.001) in all cases. Detailed signal and noise characteristics are provided in Table 5, while a pairwise comparison matrix thereof is exhibited in Table 6.

## 4. Discussion

This experimental multi-observer study investigated the feasibility and image quality of a photon-counting detector CT arthrography of the ankle joint with an ultra-high-resolution detector collimation. Employing two different dose levels and three convolution kernels with varying modulation transfer functions, the depictions of bone, cartilage, and ligaments were separately assessed. Our results indicate that bone depiction is superior in combination with an ultra-sharp reconstruction technique, whereas soft tissue evaluation benefits from employing lower spatial frequencies. As to be expected, higher noise levels and lower CNR were determined in dedicated low-dose studies; however, all assessed datasets were found to be of diagnostic quality. 

The presented findings are in line with recent publications regarding the depiction of osseous tissue with photon-counting technology in clinical applications [17,27]. As reported previously, the incorporated low-energy threshold reduces electronic background noise, which would otherwise increase significantly in low-dose applications [28]; thereby, one of the major disadvantages of a CT arthrography compared to an MRI, i.e., radiation dose, can be minimized. As the diagnostic value of CT arthrography is generally considered to be at least equivalent to an MRI after articular contrast injection [29,30], small joint imaging focused on thin layers of hyaline cartilage in particular continues to pose a major challenge to MRI arthrograms [31]. While MRI remains the modality of choice in bone marrow imaging [32,33], CT does provide advantages in assessing the subchondral bone, which facilitates diagnostic evaluation in patients suffering from osteoarthritis. Accordingly, the only previous study investigating PCD-CT arthrography reported reliable morphological assessability of cartilage loss in a porcine knee model [34].

With regards to acquisition time, an MRI also cannot compete with CT-based approaches, plausibly posing an obstacle in pain-ridden patients. As opposed to MR arthrography, the option to perform an ultrasound-guided injection of the contrast media directly within the CT suite further minimizes the overall examination time. Although a significant amount of contrast agent in the articular cavity can still be detected for up to 120 min, following the administration thereof, an acceleration of the overall procedure optimizes contrast conditions [35].

Representing a noteworthy alternative to PCD-CT arthrography of peripheral joints, with regards to achievable spatial resolution, cone-beam CT arthrography gained increasing recognition in recent years. An experimental study evaluating arthrograms of the wrist suggested superiority of a cone-beam CT approach over a conventional energy-integrating detector CT arthrography [36]. While the present investigation does not contain a direct comparison of PCD-CT and cone-beam CT arthrography, the reported dose levels for maintaining diagnostic image quality in both studies were somewhat equivalent. These findings suggest similar dose efficiencies among both techniques, mandating further investigations in patients.

The following limitations of this study ought to be considered. First, the study cohort comprised only four cadaveric specimens; however, subjective ratings were performed by seven radiologists, aiming to alleviate this restriction to some extent. Second, to offset typical drawbacks of formalin-fixated body donor studies, e.g., the deterioration of bone quality and altered soft tissue conditions, solely fresh-frozen cadavers were included. Third, due to the experimental study design, PCD-CT arthrography findings did not incur therapeutic consequences; consequently, no comparison of diagnostic performance with other imaging modalities could be drawn. Fourth, the influence of possible motion artifacts and off-center positioning on the image quality were not assessed, warranting further evaluation in a clinical patient population. Lastly, since CNR differs with radiation exposure level, the optimal kernel choice may differ for other clinical applications.

## 5. Conclusions

Photon-counting detector CT arthrography of the ankle with ultra-high-resolution collimation offers stellar image quality and tissue assessability. While bone depiction was found to be superior in combination with an ultra-sharp convolution kernel, the soft tissue evaluation benefited from employing reconstructions with a lower spatial frequency.

## Figures and Tables

**Table 1:** Acquisition protocols. Scan parameters and resulting radiation dose of photon-counting CT arthrograms.

Scan Protocol	Full-Dose Protocol	Low-Dose Protocol
Tube voltage [kVp]	120	120
Tube current-time product [eff. mAs]	125	38
Detector collimation [mm]	120 × 0.2	120 × 0.2
Pitch factor	0.5	0.5
Rotation time [sec]	1.0	1.0
CTDI_vol_ [mGy]	10	3

CTDI_vol_: volume computed tomography dose index; eff. mAs: effective milliampere-seconds; kVp: kilovoltage peak; mGy: milligray; mm: millimeter; sec: second.

**Table 2:** Reconstruction parameters. Spatial frequencies of the employed convolution kernels at different values of the modulation transfer function. Reported data according to vendor information.

Spatial Frequency	At the 50% Value of the MTF (ρ_50_) [Line Pairs/cm]	At the 10% Value of the MTF (ρ_10_) [Line Pairs/cm]	At the Maximum of the MTF (ρ_max_) [Line Pairs/cm]
Br98	39.0	42.9	20.4
Br84	22.6	27.9	10.5
Br76	16.5	21.0	7.8

Br98/84/76: vendor-specific kernel names; cm: centimeter; MTF: modulation transfer function; ρ: indicator of spatial frequency.

**Table 3:** Subjective image quality assessment. Pooled diagnostic assessability scores drawn from the subjective ratings of seven independent radiologists. Results are given as median values with 10–90 percentile ranges in parentheses.

Scan Protocol	Full-Dose Protocol			Low-Dose Protocol			ICC
Convolution Kernel	Br98	Br84	Br76	Br98	Br84	Br76	
Bone	7 (6–7)	6 (5–7)	5 (5–6)	4 (3–5)	5 (4–6)	5 (4–6)	0.938
Cartilage	6 (4–6.5)	6 (5–7)	7 (6–7)	4 (3–5)	5 (4–6)	5 (5–6)	0.887
Ligaments	6 (4.5–7)	6 (5–7)	6 (5–7)	4 (3–5)	5 (4–6)	5 (4–7)	0.805
Percentage of diagnostic examinations	100%	100%	100%	100%	100%	100%	

Br98/84/76: vendor-specific kernel names; ICC: intraclass correlation coefficient.

**Table 4:** Comparison matrix for subjective image analysis. Mean image quality ranks of protocol–kernel combinations were compared individually for bone/cartilage/ligaments in pairwise analyses.

Bone/Cartilage/Ligaments		Full-Dose			Low-Dose		
		Br98	Br84	Br76	Br98	Br84	Br76
	**Br98**		+/–/=	+/–/=	+/+/+	+/=/=	+/=/=
**Full-dose**	**Br84**	–/+/=		+/=/=	+/+/+	+/+/+	+/+/+
	**Br76**	–/+/=	–/=/=		+/+/+	+/+/+	+/+/+
	**Br98**	–/–/–	–/–/–	–/–/–		–/–/–	–/–/–
**Low-dose**	**Br84**	–/=/=	–/–/–	–/–/–	+/+/+		=/=/=
	**Br76**	–/=/=	–/–/–	–/–/–	+/+/+	=/=/=	

The Bonferroni procedure was performed to correct *p* values for multiple comparisons. “+”: superior assessability; “–”: inferior assessability; “=”: no statistically significant difference; Br98/84/76: vendor-specific kernel names.

**Table 5:** Quantitative image quality assessment. Signal and noise characteristics are reported as mean ± standard deviations.

Scan Protocol	Full-Dose Protocol			Low-Dose Protocol		
Convolution Kernel	Br98	Br84	Br76	Br98	Br84	Br76
Noise_Fat_ [HU]	149.5 ± 23.4	54.6 ± 10.6	38.9 ± 9.3	240.6 ± 56.2	78.4 ± 10.3	52.2 ± 7.5
CNR_Bone_	3.8 ± 0.9	9.8 ± 3.0	13.5 ± 4.3	3.0 ± 3.8	6.8 ± 1.6	10.2 ± 2.6
CNR_Cartilage_	3.0 ± 0.9	7.4 ± 3.5	10.2 ± 4.5	2.9 ± 4.7	5.5 ± 2.2	7.8 ± 2.3
CNR_Ligaments_	1.4 ± 3.3	3.2 ± 2.4	4.3 ± 3.4	1.2 ± 1.0	2.2 ± 1.6	2.9 ± 2.2

Br98/84/76: vendor-specific kernel names; CNR: contrast-to-noise ratio; HU: Hounsfield units.

**Table 6:** Comparison matrix for quantitative image analysis. Contrast-to-noise ratios (CNR) were calculated based on attenuation and noise measurements in the talus, talar cartilage, posterior tibiotalar ligament, and adjacent subcutaneous fat. Mean CNR ranks of protocol–kernel combinations were compared individually for bone/cartilage/ligaments in pairwise analyses.

CNR_Bone_/CNR_Cartilage_/CNR_Ligaments_		Full-Dose			Low-Dose		
		Br98	Br84	Br76	Br98	Br84	Br76
	**Br98**		–/–/–	–/–/–	=/=/=	–/–/–	–/–/–
**Full-dose**	**Br84**	+/+/+		–/–/–	+/+/=	+/+/+	=/=/=
	**Br76**	+/+/+	+/+/+		+/+/+	+/+/+	+/+/+
	**Br98**	=/=/=	–/–/=	–/–/–		–/=/=	–/–/=
**Low-dose**	**Br84**	+/+/+	–/–/–	–/–/–	+/=/=		–/–/=
	**Br76**	+/+/+	=/=/=	–/–/–	+/+/=	+/+/=	

The Bonferroni procedure was performed to correct *p* values for multiple comparisons. “+”: higher contrast-to-noise ratio; “–”: lower contrast-to-noise ratio; “=”: no statistically significant difference; Br98/84/76: vendor-specific kernel names; CNR: contrast-to-noise ratio.

## Data Availability

The datasets generated and/or analyzed during this study are not publicly available as CT data and DICOM headers contain patient information. Data can be obtained upon reasonable request from the corresponding author.

## Abbreviations

CNR	contrast-to-noise ratio
CTDI_vol_	volume computed tomography dose index
PCD	photon-counting detector
UHR	ultra-high-resolution
